# The effect of disease burden on the speed of aging: an analysis of the Sardinian mortality transition

**DOI:** 10.1186/s41118-018-0028-8

**Published:** 2018-08-01

**Authors:** Giambattista Salinari, Gabriele Ruiu

**Affiliations:** Department of Economics and Business, University of Sassari, Via Muroni 25, 07100 Sassari, Italy

## Abstract

According to the *constant senescence hypothesis*, senescence cannot be accelerated or decelerated by exogenous factors. Two contrasting theories have been proposed in the literature. According to the *inflammaging theory*, those individuals who have experienced a higher antigenic load will experience more rapid senescence. Instead, the *calorie restriction* theory stresses that excessive daily calorie intake can produce an acceleration in senescence.

To test these theories, this paper analyzes the evolution of the rate of aging in Sardinia (Italy). In this population, the epidemiological transition started without any substantial modification in nutritional levels. This allows us to test the constant senescence hypothesis against the inflammaging theory, without the possible confounding effect produced by the nutrition transition.

To accomplish this aim, the longitudinal life tables from 80 years onwards for Sardinian cohorts born between 1866 and 1908 were reconstituted. They were then used to estimate the rate of aging by means of the Gamma-Gompertz model. Coherently with the inflammaging theory, the results show that the Sardinian population experienced a dramatic decrease in the rate of aging that coincided with the onset of the epidemiological transition.

## Introduction

In biology, the term *senescence* is used to indicate the progressive accumulation of molecular damage that takes place in an organism as time goes by (Rattan 2006, 2008; Yin and Chen 2005). In biodemography, the analysis of senescence is indirectly carried out through the examination of the effect that this process produces on mortality (Finch 1994; Vaupel 2010). Indeed, senescence triggers a series of deleterious effects (e.g., sarcopenia, immuno-senescence, systemic inflammation, the onset of degenerative diseases, etc.). These result, starting from a certain age (aging onset), in a gradual growth in the risk of death (*demographic aging*). Thus, by studying the pace at which mortality accelerates, it is possible to infer the general characteristics of the senescence process and to investigate which factors accelerate/decelerate its progression.

So far, three major explanations for the determinants of senescence have been proposed: the *constant senescence hypothesis*; the *inflammaging theory*; and the *calorie or energy restriction theory*.

According to the constant senescence hypothesis (Vaupel 2010), the pace of senescence is a biological constant among humans. As a result, senescence cannot be accelerated or decelerated by exogenous factors.

Instead, the inflammation theory (Franceschi et al. 2000) claims that the number and intensity of immune system responses to antigenic load in a lifetime is a fundamental factor in regulating the pace of senescence. Thus, individuals who have experienced a higher exposure to antigenic load will also experience a more rapid aging process.

Finally, the calorie restriction theory (Masoro 2005), which is based on a plethora of experiments on a vast range of mammals and non-mammals, explores a reduced daily calorie intake and its positive effect on aging. In particular, a reduction in daily calorie intake is thought to slow senescence.

From a theoretical point of view, the three theories can be empirically tested. This would require a comparison of the aging process in cohorts who have experienced different nutritional regimes and different disease loads. However, the coincidence of the epidemiological transition (Omran 2005) with the onset of the nutrition transition, from a low to a high calorie regime (Grigg 1995; Popkin 1993), makes it difficult to isolate the effects of these two contrasting forces on mortality acceleration.

The epidemiological transition in Sardinia is unusual in that it started without any substantial modification in nutritional levels. This makes Sardinia a quasi-natural experiment where we can test the constant senescence hypothesis against the inflammation theory, without the confounding effect of changes in nutritional levels.

To implement the analysis, the longitudinal life tables from 80 years onwards for Sardinian cohorts born in the period 1866-1908 were reconstituted and used to estimate the Gamma-Gompertz model: this model assumes that the individual hazard function follows the Gompertz model and that frailty is Gamma-distributed. The β parameter of the Gamma-Gompertz model, the so-called rate of aging, measures the relative derivative of the force of mortality, and in this sense, it may be used to measure how fast mortality progresses with age.

The results show that the Sardinian population experienced a dramatic reduction in the rate of aging that coincides with the onset of the epidemiological transition.

The structure of this paper is as follows: in the following section, there is a brief overview of the three theories given above; the third and the fourth sections describe, respectively, the Sardinian epidemiological and nutrition transition. The fifth section presents the methodology used to test the constant senescence hypothesis against the inflammation theory, while associated results are shown in following section. The last section is devoted to final remarks.

## Three hypotheses on the determinants of aging

The three hypotheses covered in this work stem from different scientific disciplines and different kinds of empirical evidence: experiments on animal models, clinical observations and life tables.

The constant senescence hypothesis has recently been advanced in demographic literature by Vaupel (2010) and, while it refers to individual mortality, it has been primarily tested using aggregate data from life tables. A rough constancy in the rate of aging has been observed, both from a cross-sectional point of view (i.e., for different populations) and from a historical point of view (i.e., for different time periods). For example, the analysis carried out by Gurven and Kaplan (2007) on various populations of hunter-gatherers highlights that mortality rate doubling time (from hereon, MRDT) varies from about 6 to 9 years. In the case of nineteenth-century European populations (France, Italy, Sweden) (Barbi 2003; Barbi et al. 2003; Finch 1994; Finch et al. 1990) and contemporary economically developed societies (Bronikowski et al. 2011 for the USA), the MRDT is estimated at around 7 to 8 years. Hence, despite huge changes in the levels of mortality and life conditions, the MRDT seems to remain roughly stable.

An important piece of evidence in favor of the constant senescence hypothesis comes from the recent discovery of the old age mortality plateau (Gampe 2010). It is known that death probability leveling-off at very old age (between 110 and 120 years) is, in fact, inconsistent with the class of accelerated failure time models (Finkelstein and Esaulova 2006; Missov and Vaupel 2015), which assume that the rate of aging can vary across different individuals.

The inflammation theory was developed in gerontology (Franceschi et al. 2000, 2007) and is mainly based on clinical data. The starting point for this theory was the empirical observation that a systemic inflammation is often associated with aging (inflammaging) and the onset of chronic and degenerative diseases, as well as with the acceleration of death probability. According to Baylis et al. (2013), inflammaging derives from a cumulative lifetime exposure to antigenic load. In particular, each time the immune system reacts to aggression, it also produces irreversible cellular damage to tissues and organs, which once a triggering threshold has been reached, takes on a chronic proinflammatory state. This, in turn, has been shown to increase susceptibility to age-related diseases/disabilities.

From a biodemographic point of view, inflammaging has two main theoretical consequences.

Firstly, it implies the presence of cohort effects: the higher the exposure to infections for a cohort, the higher the level of inflammation and, consequently, of mortality (Finch and Crimmins 2004). On empirical grounds, analysis has found mixed results. Bengtsson and Lindström (2000, 2003) and Bengtsson and Broström (2009) were able to find cohort effects in Sweden. However, Gagnon and Mazan (2009) did not confirm this result for French Canada.

Secondly, inflammaging means that those who experience higher exposure to antigenic load, especially in their infancy, will also be characterized by more rapid aging (De Martinis et al. 2005). Recent research works on two “not-westernized” contemporary populations (Gurven et al. 2009; Koopman et al. 2012) exposed to high disease-load found, however, that both populations are characterized by a lower prevalence of cardiovascular risk-factors (hypertension, diabetes, total cholesterol etc.) than European and US populations.

This lack of concordance between empirical analyses may be caused by the neglect of the role of nutrition on aging (see below).

The calorie restriction theory was developed in the ambit of biogerontology in experiments conducted on a vast range of animal species. In particular, the experiments on calorie restriction have been carried out on yeast (Jiang et al. 2000), fruit-flies (Bross et al. 2005; Mair et al. 2003), nematodes (Lenaerts et al. 2007; Yen and Mobbs 2008; Houthoofd et al. 2002), crustaceans (Ingle et al. 1937), spiders (Austad 1989), rodents (see Masoro 2005, 2009 for a review), and non-human primates (Bodkin et al. 2003; Colman et al. 2009, 2014; Mattison et al. 2012). In very recent times, there has also been experimentation on human volunteers (see for a review Bartke 2012; Holloszy and Fontana 2007; Longo and Fontana 2009; Roth and Polotsky 2012).

Usually, in calorie restriction experiments, nutrition is reduced after weaning in the treatment group to about 30–60% of the normal (ad libitum) level and is, then, maintained lifelong at this level. At the same time, the control group is left free to feed to repletion. For both the control and the treatment group, the experiment is carried out in a pathogen-free environment. This allows for the isolation of the effect of nutrition from that of infections. Most of the experiments confirm that the treatment group enjoys a 30–60% gain in both average lifespan and maximum lifespan (Omodei and Fontana 2011). This higher longevity stems from a lower incidence of degenerative diseases. Furthermore, the treatment group is also characterized by a lower level of inflammation, a lower level of free-radicals, the delayed onset of aging and slower mortality acceleration. The onset of aging is measured in these experiments by the age when the first deaths from senescent causes take place, such as cancer, heart, and kidney failure, strokes etc.

Some variants of this experimental design have tried to investigate the effects of calorie restriction when the latter is initiated in adulthood. In this case, the treatment appears less effective, with an increase of 10–20% in terms of average and maximum lifespan (Omodei and Fontana 2011).

Finally, other kinds of experiments focus on the reactions of both the treatment and control group to antigenic load. In particular, it is shown that when experimental subjects are inoculated with viruses (Gardner 2005; Ritz and Gardner 2006; Ritz et al. 2008), bacteria (Sun et al. 2001) or parasites (Kristan 2007), the treated group appears less capable of fighting external aggression than the control group.

The last findings point out a non-intuitive feature of the relationship between nutrition and mortality. In a non-pathogen-free environment, like the one in which we normally live, better nutrition entails a beneficial effect on the general level of mortality (because it helps to counter infections), but a detrimental effect on the rate of aging.

**Fig. 1:**
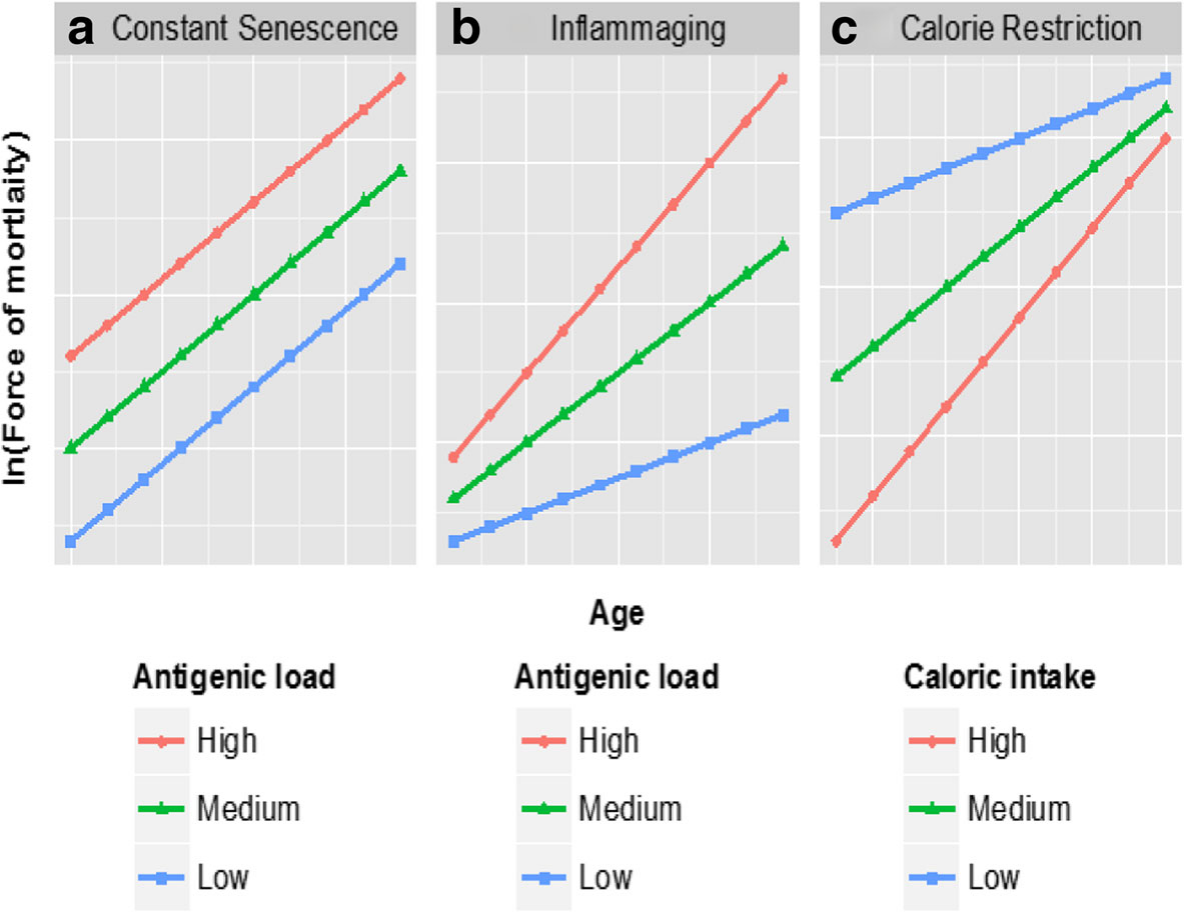
Three hypotheses on the determinants of senescence. *Note*: The curves represent the evolution of the individual force of mortality after the aging onset. *Sources*: Authors’ simulation

Figure 1 summarizes the predictions of the three theories described here. To recap, the aim of this paper is to test the hypothesis represented in Panel a (constant senescence) against that reported in Panel b (inflammaging), without confounding effects from nutrition (Panel c). As we will show in the next section, Sardinia is an ideal candidate for looking at this question.

## The epidemiological transition in Sardinia

In recent years, scholars interested in aging have turned, with increasing interest to, Sardinia. This is because of the particularly high number of centenarians who live today on this island (Caselli et al. 2006, 2013; Pes et al. 2013; Poulain et al. 2013). However, before being known as the island of centenarians, Sardinia was known as an island of malaria. Sardinia was plagued, indeed, by this disease from the first millennium B.C. and malaria is mentioned in the writings of Roman writers of both the republican and imperial age (Sallares 2002). This disease was so omnipresent in Sardinian life that it produced several genetic adaptations in the Sardinian population (Gatti and Puggioni 1988).

The main feature of malaria is to establish a long-run equilibrium with the host organism. After the initial stage of infection—during which, the risk of dying is higher—the individual suffers periodical febrile reactions, that last for three or four days (depending on the species of Plasmodium). After several re-infections, individuals can develop partial immunity (Doolan et al. 2009). Thus, malaria periodically stresses the immune system for a long period of time, which, according to the theory of inflammation, should result in faster aging.

The first official statistics of the Italian Kingdom (Ministero Agrigoltura, Industria e Commercio 1890) on the causes of death have Sardinia as the Italian region in which malaria was responsible for the highest percentage of deaths. Each year, about 10% of Sardinian deaths were attributable to malaria, which made this disease the first cause of death there. In addition to the direct lethal effects of malaria, this disease allowed other pathogens much easier access to the host organism. Malariologists estimate that malaria is effectively responsible for about three times more deaths than those directly attributed to the disease (Sallares 2002:121). If this rule of thumb is applied to Sardinia, about one in three Sardinian deaths were directly or indirectly attributable to malaria in the second half of the 19th century.

**Fig. 2:**
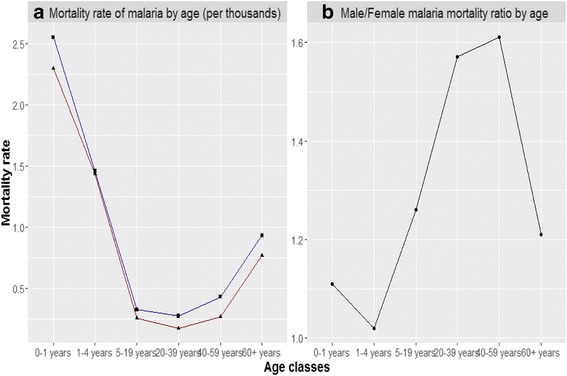
Evolution of malaria mortality by age and gender. *Note:* Panel **a** shows the evolution of malaria mortality by age and gender. The blue line indicates the mortality rate for males, the red line indicates the mortality rates for females. Panel **b** shows the Male/Female mortality ratio by age. *Source*: Authors’ calculation on Ministero Agricoltura, Industria e Commercio (1890) data

The pattern of malaria mortality by age shows that the highest number of deaths was concentrated in the early years of life. For 5 to 19 years, mortality fell to a rate of about 0.025 per thousand, while it rose again in the older age brackets (see Fig. 2). The male population was most affected. This is likely due to the fact that males were exposed to anopheles mosquitoes (the vector of the disease) while working in the fields.

**Fig. 3:**
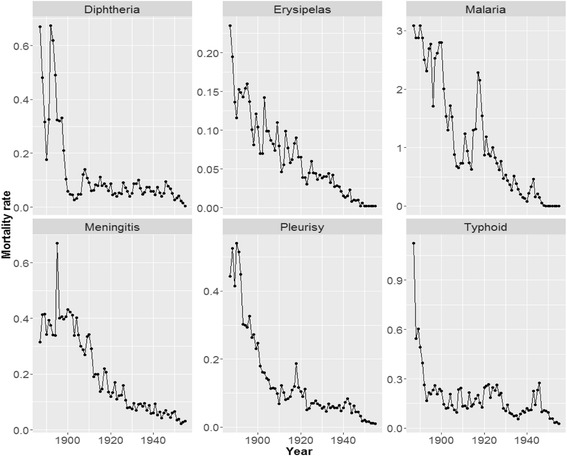
Some aspects of the epidemiological transition in Sardinia. *Note:* Mortality rate are expressed as deaths per 1000 average year population. *Source*: Authors’ calculation on Istat (1958) data

A major innovation was introduced in 1900 with law nr. 505/1900 (known as the “legge del chinino di Stato”). This forced all municipalities and landowners to bear the local costs of the quinine prophylaxis. The temporal evolution followed by mortality due to malaria shows the effectiveness of this legislation (see Fig. 3). After this legislative innovation, deaths caused by malaria began to decline rapidly (Corti 1984; Tognotti 1996). However, in terms of morbidity, the effects of the law were less satisfactory. All available sources indicate that, in the 1930s, 47% of the population was still infected by this disease, even if the mortality due to malaria had declined almost to nothing in Sardinia. This suggests that quinine was used to defend the most fragile part of the population, namely children in the 0–10 age bracket (those most vulnerable to malarial deaths). At the same time, its use was probably considered unnecessary for adults who had already been partly immunized.

The “chinino di Stato” law, though probably the most important and most effective sanitary intervention of the young Italian State, was by no means unique. Early measures for improving hygiene conditions were introduced during the cholera epidemic of 1884–85 (Forti Messina 1984). These measures, which encouraged Italians to wash hands and boil water, almost eliminated the disease from Italy. Furthermore, these measures were probably responsible for the reduction in the incidence of typhoid fever (Faccini 1984; Tognotti 2008). In 1888, the smallpox vaccine was made compulsory with the “Crispi-Pagliani” law (Tucci 1984). The use of the serum against diphtheria and tetanus dates to approximately the same epoch (Tognotti 2008).

Overall these sanitary interventions (see Fig. 3) mark the 1890s as a turning point in the epidemiological history of Sardinia. Confirming this timing of the epidemiological transition, Ruiu (2017) shows that the introduction of both the Crispi-Pagliani Law and the Chinino Law were associated to sensible changes in the seasonal pattern of deaths, suggesting that the underlying epidemiological regime was thus changing. According to the inflammation theory, the cohorts born before the last decade of the 19th century should have experienced a faster aging process than the cohorts born subsequently.

## The nutrition transition in Sardinia

During the 1930s and, therefore, before the end of the old Sardinian demographic regime (Gatti and Puggioni 1988), Sardinia was studied by nutritionists, interested in determining the differing nutritional levels of the Italian regions (see Somogyi 1973 and Vecchi 1994). These are the first Italian surveys where food consumption is directly observed at the household level. Furthermore, in these investigations, consumption was broken down into carbohydrates, proteins, and fats by methods that resemble modern nutritional studies (Zamagni 1998). Among these studies, the most complete and rigorous investigation was probably that carried out in 1939 by Peretti (1943) on three villages in the Nuoro district (central Sardinia, see Table 1b). The adult male peasant population of the three villages had an average height of 157 cm and an average weight of about 52-53 kg. The resulting body mass index (BMI) was, thus, of about 21 kg/m^2^. It is worth noting that the literature on calorie restriction indicates that a body mass index of 21 kg/m^2^ is the threshold value under which a significant increase in longevity should be expected (Omodei and Fontana 2011).

**Table 1:** Sardinia before the Nutrition Transition

*(a) % Male population active in agriculture*									
Authors			1881	1901	1911	1921	1931	1951	1961
Censuses – rural population (%)			62.3	67.8	63.8	64.9	63.5	55.7	37.5
*(b) Surveys on Sardinian nutrition during the 1930s*									
Authors and year of investigation	Occupation	District	Proteins		Carbohydrates		Lipids		Calorie
			g	cal. %	g	cal. %	g	cal. %	kcal
Niceforo and Galeotti (1934)	Farmhands	Sassari	89.0	13.7	444.0	68.5	52.0	17.8	2656.0
Niceforo and Galeotti (1934)	Agricultural worker (small Landowner)	Sassari	103	13.5	477	64.9	71	21.3	3024.1
Cao-Pinna (1935)	Craftsman	Cagliari	79.0	14.5	359.9	66.3	46.9	19.2	2225.9
Costanzo (1937)	Labourer	Cagliari	–	14.3	–	71.0	–	13.2	2887.0
Peretti (1939)	Agricultural worker	Nuoro (Desulo, Atzara, Sorgono)	116.0	17.3	460.0	68.6	43.0	14.2	2752.5
Peretti (1939)	Shepherd	Nuoro (Desulo, Atzara, Sorgono)	119.0	18.6	406.5	62.8	54.0	18.7	2645.5

*Note*: In Panel (b), the reported year is that in which the surveys have been carried out (not the year of publication of the results by the authors). In Panel (c), CU indicates urban population and CR indicates the rural population

*Sources*: Niceforo and Galeotti (1934); Cao-Pinna (1935); Costanzo (1940); Peretti (1943); Istat − 1944 (cited in Somogyi 1973)

The diet followed by the peasant population was practically vegetarian, with only the occasional consumption of meat, mostly tied to feasts and celebrations. The daily calorie consumption for both farmers and shepherds amounted to about 2600–2800 k-calories/day (excluding wine consumption).

Other estimates, conducted in the 1930s by different authors in different areas of Sardinia, seem to be, at least in part, consistent with Peretti’s data. The study conducted by Niceforo and Galeotti (1934) on the small landowners of Sassari (in the Northern part of the island) gave an average daily caloric intake of about 3000 k-calories, while the caloric intake for the daily laborers in the same area was about 2700 k-calories *per* day. A study conducted by Cao-Pinna (1935) on craftsmen of Cagliari (Southern part of the island) reports a lower caloric intake: about 2300 k-calories *per* day. Finally, the analysis carried out by Costanzo (1940) on industrial workers in Sassari in 1937, reports a daily caloric intake of about 3000 k-calories.

Consequently, it seems likely that in the 1930s the vast majority of the Sardinian population consumed an average of 2700 ± 400 kcal *per* day. This is about 10% less than the 3000 k-calories, calculated by Federico (2003) in 1910 for Italy using data derived from the national accounts). It is also lower with respect to the caloric intake estimated for other Italian regions (see, for instance, Table 11 in Peretti 1943).

**Fig. 4:**
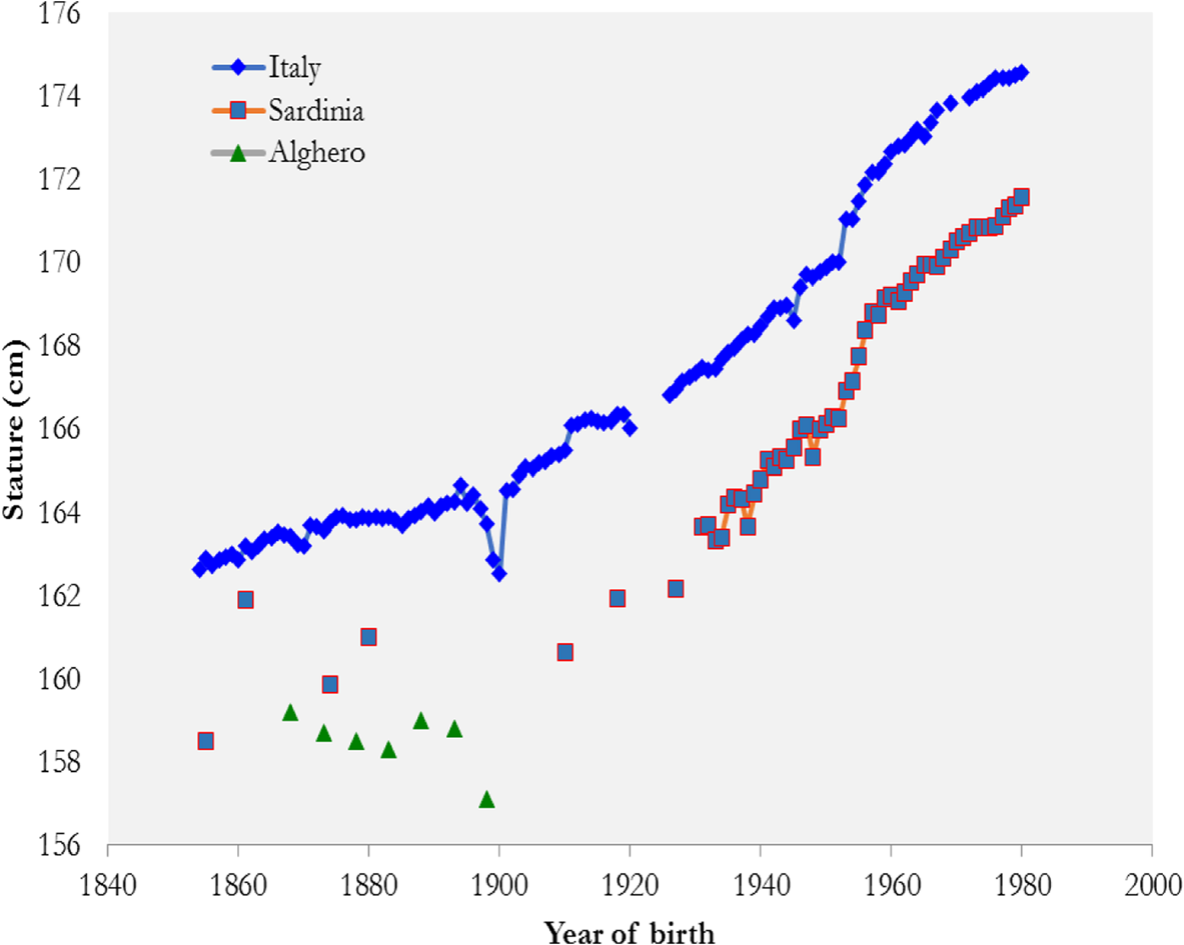
Evolution of heights in Italy and Sardinia. *Note*: Alghero is a town in the North-West of Sardinia

The data on statures (Fig. 4) confirm the above. Together with Basilicata, Sardinia was the Italian region with the lowest average height (A’Hearn et al. 2009; Arcaleni 1998, 2006; Sanna 2002; Sanna et al. 1993). In particular, data from the conscript records reveal that the average of the Sardinian cohorts born between 1861 and 1927, ranged at 20 years of age, between 160 and 162 cm, without any substantial upward trend in the time series. Conversely, the Italian population shows, in the same epoch, an upward trend in average height from approximately 163 cm for the 1861 cohort to 167 cm for the 1927 cohort. This process led to a progressive divergence between the stature of Sardinian conscripts and those from other Italian regions (see also Mazzoni et al. 2017, for a discussion of the link between low nutritional levels and the height of the soldiers coming from Alghero, a Sardinian coastal town in the north-west, at the turn of the 20th century).

Sardinia saw a steeper increase in average heights than Italy, beginning, according to military records, with cohorts born in the 1930s. This evidence, therefore, seems to indicate that the period between the 1930s and 1950s marked the onset of the nutritional transition on the island: the cohorts born in 1930s would have been measured 20 years later.

## Data and methods

**Fig. 5:**
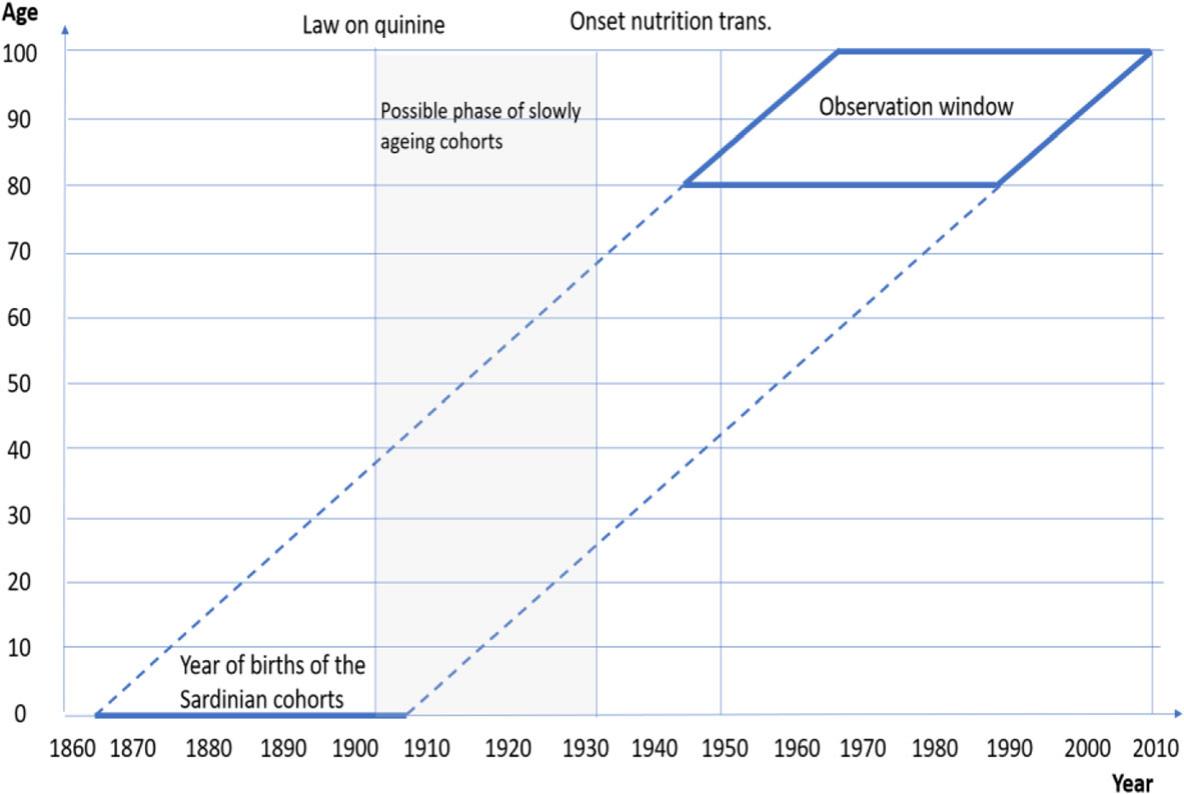
Data description

The available studies on the nutritional level and on average cadet height seem to suggest that Sardinian cohorts born between 1860 and 1930 did not experience any significant increase in their nutritional levels. Therefore, one may assume that the aging process had not, in this period, been influenced by the, possibly detrimental, passage to a modern nutritional regime. However, the sanitary interventions of the new Italian State, in the 1890s, made the Sardinian population healthier. Thus, the cohorts born between then and 1930 are ideal for a quasi-experiment. If the inflammation theory is correct one should observe, for the cohorts born between the 1890s and 1930s, a marked fall in the rate of aging. Conversely, if the constant senescence hypothesis is true no significant difference in the rate of aging should emerge for cohorts from this period and before (see Fig. 5).

In order to gauge the rate of aging for the cohorts born before and after the onset of the epidemiological transition the longitudinal life tables for the Sardinian population aged 80+ years have been reconstituted. This has been done following the indications proposed in the *Methods Protocol for the Human Mortality Database,* available on the web site of the Human Mortality Database (HMD) (www.mortality.org) harmonizing the Sardinian data with that of the HMD. The raw data for our reconstitution are represented by the cross-sectional death counts classified by age and sex published by ISTAT for the different Italian Regions. The reconstitution of the longitudinal life tables was facilitated by the fact that Sardinia, being an island, has not changed its administrative boundaries in the last 150 years. To reconstitute the longitudinal (by cohort) death counts the cross-sectional death counts were first split into Lexis triangles. The life table for those who survived to 80 was then computed through the extinct-cohort method.

Eighty years was chosen as the starting point for the calculation of the Sardinian Life tables to reduce any bias from migration.

The present analysis begins with the Sardinian cohort born in 1866 which figures in our observations 80 years later, in 1946, after the Second World War. The last cohort covered by the analysis is that born in 1908. Thus, the youngest cohorts (those born after the 1890s) analyzed in this paper are those who experienced the initial reduction in the disease load (see the third section), but who did not benefit, during their childhood, from substantial nutritional improvements (see fourth section).

All reconstituted cohort life tables range from 80 to 110 years, but the estimates of the rate of aging was carried out on the 80-99 years age interval. This avoids any bias introduced by the frequent use in the raw data of a terminal open age class 100 + .

To estimate the rate of aging for these cohorts, the Gamma-Gompertz model was employed (Vaupel et al. 1979). This model is based on four hypotheses (Yashin et al. 2000): individual frailty follows a Gamma distribution; individual frailty does not change as a function of age; the individual hazard function follows the Gompertz law; and the rate of aging is constant across different individuals. From these assumptions, the log aggregate force of mortality ${\overline{\mu}}_x$ for a single cohort *j (j = 1866, 1867, …, 1908)* at age *x* is (Vaupel et al. 1979):

1 $$ {\overline{\mu}}_x=\alpha +\beta x+{\sigma}^2{\overline{s}}_x+{\epsilon}_x. $$

where ${\overline{s}}_x$ indicates the log aggregate survival function and *α*, *β*, and *σ*^2^ are the three parameters of the model. The *α* parameter represents the logarithm of the initial mortality of the cohort, *β* indicates the rate of aging, and *σ*^2^ represents the initial frailty variance. Note that both the *β* and the *σ*^2^ parameters are expected to be positive values.

The Gamma-Gompertz model is taken as the reference model for the analysis of demographic aging. In addition to its mathematical tractability, which has undoubtedly favored its use, various empirical analyses have shown that the fitness of Gamma-Gompertz to empirical hazard functions is better than that obtained with alternative models (Manton et al. 1986; Yashin et al. 1994).

Furthermore, each of the three theoretical predictions from this model—the process of mortality deceleration, the formation of a mortality plateau and the processes of mortality cross-over—have found support on empirical grounds (Gampe 2010; Manton and Stallard 1981; Manton and Vaupel 1995; Vaupel et al. 1998). For this reason, Missov and Vaupel (2015) concluded that the Gamma-Gompertz model is the only demographically meaningful multiplicative model which is compatible with the observed leveling-off of human mortality at 110+ years.

Our first analysis consisted in estimating Eq. (1) using one life table (cohort) at a time. In this way, we performed 43 independent estimates of Eq. (1), separately for males and females, one for each cohort born in 1866–1908. The series of estimated parameters allowed us to assess the overall evolution followed by the three parameters (*α*, *β*, and *σ*^2^) of the model throughout the period under analysis. The fitting procedure for Eq. (1) assumes that the deaths *D*_*x*_ at age x are Poisson distributed (Brillinger 1986). Under such an assumption the log-likelihood, ln*L*, for the model emerges as:

2 $$ \ln L\left(\alpha, \beta, {\sigma}^2\right)={\sum}_x\left[{D}_x{\mu}_x-{E}_x\mathit{\exp}\left({\mu}_x\right)\right]. $$

Here *E*_*x*_ indicates the exposures at age x. The parameters estimate has been performed through generalized linear models (Poisson regression), and through an iteratively-reweighted least-squares optimization (Mccullagh and Nelder 1989).

In order to formally test for the constant senescence hypothesis, we first pooled together all the life tables (cohorts) in our database and we proceeded to estimate the mean rate of aging for all cohorts in the dataset. This may be done by estimating the following model:

3 $$ {\overline{\mu}}_{j,x}={\alpha}_j+\beta x+{\sigma}_j^2{\overline{s}}_{j,x}+{\epsilon}_{j,x}, $$

Cohorts were then broken down into five non-overlapping groups: 1866–1869, 1870–1879, 1880–1889, 1890–1899, 1900–1908. The rate of aging was, this time, estimated in each group by introducing in Eq. (3) a set of dummy variables (*g*_k_), whose value is 1 for the group of cohorts of interest and 0 otherwise:

4 $$ {\overline{\mu}}_{j,x}={\alpha}_j+{\sum}_{k=1}^4{\mathrm{g}}_k{\upbeta}_k\mathrm{x}+{\sigma}_j^2{\overline{s}}_{j,x}+{\epsilon}_{j,x}. $$

By estimating Eq. (4) on the entire set of cohorts (from 1866 to 1908), it becomes possible to verify whether significant differences exist in the rate of aging experienced by the different groups of cohorts. Conversely, by estimating Eq. (3) on the entire set of cohorts, one gets the average rate of aging experienced by all the cohorts during the whole period. These two nested models can be compared with an *F* test or a likelihood ratio test: if the proportion of variance explained by Eq. (4) turns out to be significantly greater than that explained by Eq. (3), then the rate of aging has undergone a significant change over the period under scrutiny, and the constant senescence hypothesis should be rejected.

Equations (3) and (4) are theoretically prone to the incidental parameter problem, because of the presence of the cohort specific parameters α_j_ and ${\upsigma}_{\mathrm{j}}^2$ (Neyman and Scott 1948). However, in a recent article it has been shown that this bias is negligible (Salinari and De Santis 2014).

Another possible problem with Eqs. (3) and (4) is represented by the fact that they do not take into account the potential bias over the rate of aging due to period effects. As we have seen, the Sardinian cohorts born during the period 1866–1908 reached 80 years of age (and enter observation) during the period 1946–1988. The period from the 1970s onward is known to be characterized by the introduction of several medical and sanitary innovations, which had a considerable effect on mortality among the elderly (Deaton 2013). These innovations are thought to influence the mortality rates in a cross-sectional way. This means that, since the introduction of these medical innovations, all cohorts in the population will benefit from the innovation. The existence of this kind of effect represents a problem for our analysis, because period effects may result in a bias of the estimates in the rate of aging (Salinari and De Santis 2014). In other words, a sudden downward shift in the mortality hazard function may be confused and wrongly interpreted in our estimation of Eqs. (1), (3), and (4) as a decrease in the rate of aging. To protect the analysis from this kind of bias, a possible solution would be to include in the model a set of dummy variables (time fixed effects) aimed at capturing the downward shift produced by medical outbreaks. From a theoretical point of view, this approach may be criticized because it assumes that period effects (the benefits produced by medical outbreaks) are independent of age. A different way of getting rid of period effects, which also takes into account that period effects may differ by age, consists in differentiating Eqs. (3) and (4) with respect to age:

5 $$ \Delta  {\overline{\mu}}_{j,x}=\beta +{\sigma}^2\Delta  {\overline{s}}_{j,x}+{\nu}_{j,x}, $$

6 $$ \Delta {\overline{\mu}}_{j,x}={\sum}_{k=1}^4{\mathrm{g}}_k{\upbeta}_k+{\sigma}^2\Delta {\overline{s}}_{j,x}+{\nu}_{j,x}. $$

where $\Delta  {\overline{\mu}}_{j,x}={\overline{\mu}}_{j,x}-{\overline{\mu}}_{j,x-1}$ and $\Delta  {\overline{s}}_{j,x}={\overline{s}}_{j,x}-{\overline{s}}_{j,x-1}$. This approach is quite standard in panel data analysis (Wooldridge 2002). The central hypothesis is that period effects are virtually the same at two contiguous ages *x* and *x* + 1, so that their effect is eliminated when mortality is differentiated with respect to age. This approach presents two further advantages with respect to time fixed effects. First, it reduces the number of parameters to be estimated. Second, since differentiation removes the *α* parameters from the model, we can exclude the possibility that a correlation between the *α* and *β* parameters (Burger and Missov 2016:36) causing bias in our estimates (indeed, through differentiation, we get rid of the intercept of the model). The most important drawback is, instead, represented by the fact that, as a consequence of differentiation, the noise affecting our data doubles. Indeed, according to Brillinger (1986), the sample variance of ${\overline{\mu}}_x$ is $\frac{1}{D_x}$. Assuming independence between ${\overline{\mu}}_{x+1}$ and ${\overline{\mu}}_x$ we can conclude that the sample variance of $\Delta  {\overline{\mu}}_x$ is $\frac{1}{D_{x+1}}+\frac{1}{D_x}$. Since *D*_*x* + 1_ and *D*_*x*_ have similar values, the sample variance of $\Delta  {\overline{\mu}}_x$ can be approximated as $\frac{2}{D_x}$. This means that Eqs. (5) and (6) cannot be estimated through a Poisson regression because of over-dispersion. For this reason, we resolved to use, in this case, weighted least squares (WLS) with weights equal to half the number of deaths *D*_*x*_ (*x* = 80, 81, …, 99).

Since one reason for focusing on Sardinia, is the unusual timing of the epidemiological/nutritional transition there, it seems natural to compare the evolution of the rate of aging in the island with that of Italy more generally. To this end, we employed the lifetables of the Italian cohorts in the Human Mortality Database (HMD) for Italy, and carried out the same kind of analyses performed for Sardinia. However, since the data in the HMD start with the Italian cohort born in 1872, we were obliged to restrict the analysis to the Italian cohorts born between 1872 and 1908. To assess whether the rate of aging in Sardinian was different from that of Italy as a whole, we first pooled together the differentiated log hazard function for the Italian and Sardinian cohorts in a common dataset. Then, we inserted, in Eq. (5), a dummy variable (region fixed effect) aimed at capturing the possible difference in the rate of aging experienced by the cohorts belonging to these different contexts:

7 $$ \Delta  {\overline{\mu}}_{j,x}={\beta}_1+{\beta}_2{\xi}_{j,x}+{\sigma}^2\Delta  {\overline{s}}_{j,x}+{\nu}_{j,x}. $$

In Eq. (7), the *ξ*_*j*, *x*_ thus represents a dummy variable whose value is 1 for the Sardinian differentiated log hazard and 0 otherwise. In particular, we used Eq. (7) to compare the value of the rate of aging in Italy and Sardinia before the advent of the epidemiological transition.

## The evolution of aging in Sardinia

**Fig. 6:**
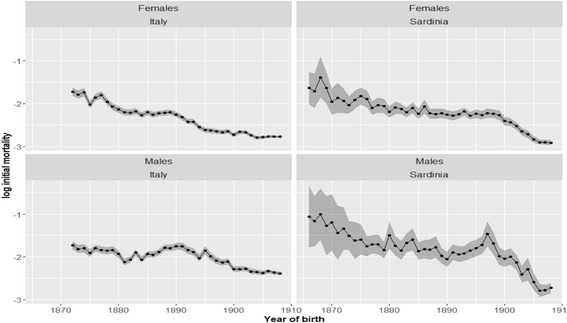
Estimated initial mortality. *Note*: Each point represents the estimate of the *α* parameter of Eq. (1) for a given cohort. *Source*: ISTAT and HMD

**Fig. 7:**
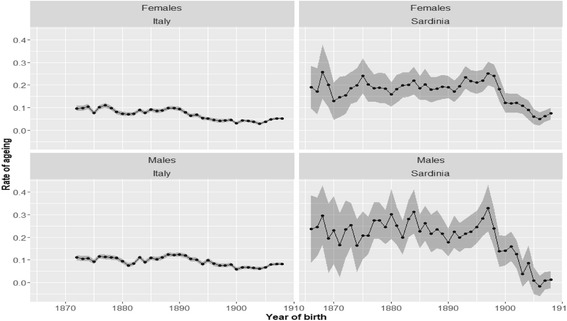
Estimated rate of aging. *Note*: Each point represents the estimate of the *β* parameter of Eq. (1) for a given cohort. *Source*: ISTAT and HMD

**Fig. 8:**
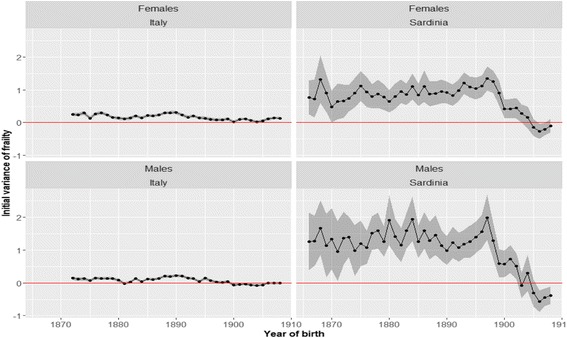
Estimated initial variance of frailty. *Note*: Each point represents the estimate of the *σ*^2^ parameter of Eq. (1) for a given cohort. *Source*: ISTAT and HMD

**Fig. 9:**
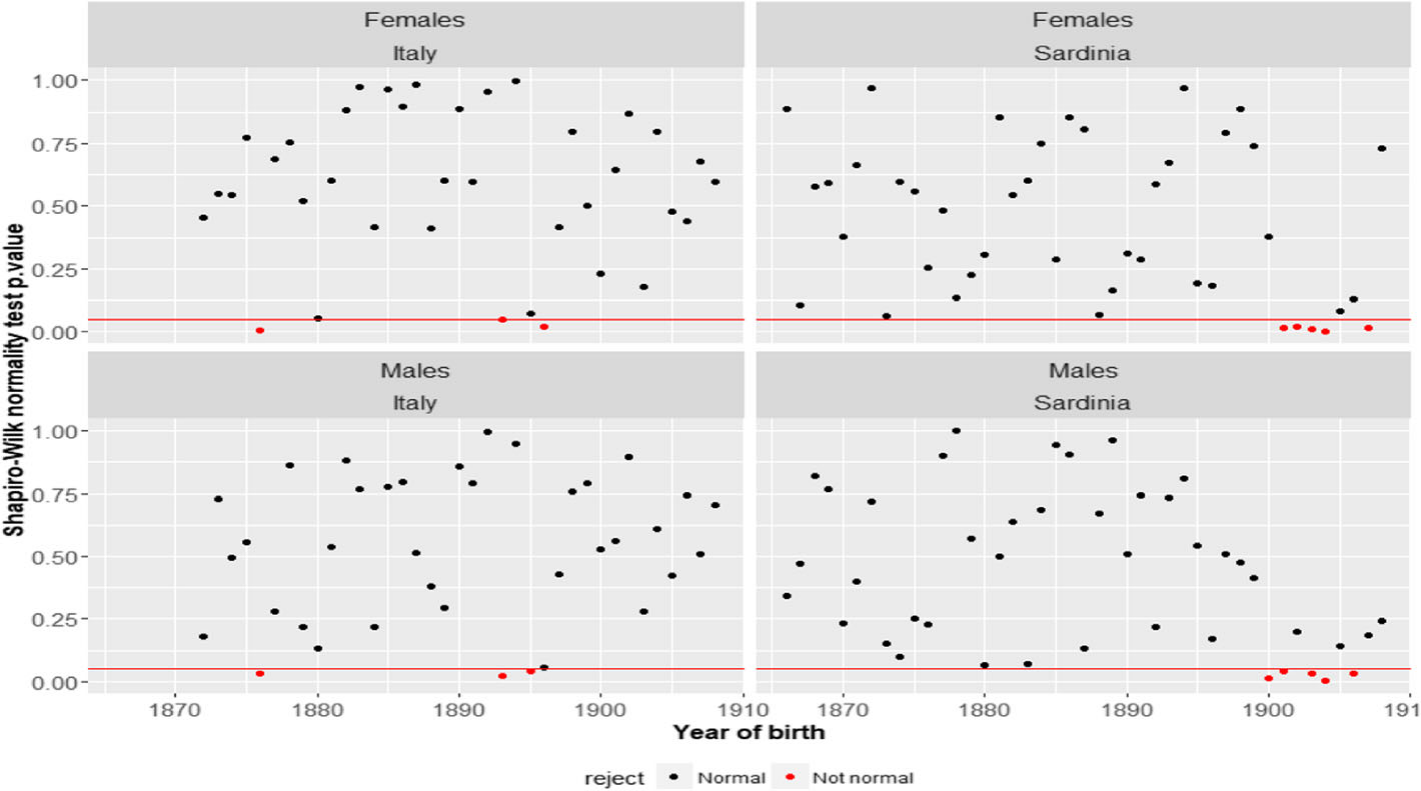
Models diagnostic. *Note*: Each point represents the *p* value associated with the Shapiro-Wilk normality test. The (red) points below the threshold of 0.05 (red horizontal line) indicate those cases when the hypothesis of normality is rejected. *Source*: ISTAT and HMD

To analyze the evolution over time of the rate of aging in Sardinia, we first estimated the Gamma-Gompertz model one cohort at a time for the year of birth, 1866–1908. The model was estimated through Poisson regression, separately for the Sardinian and for the Italian cohorts, and separately for males and females. The results of this analysis along with some diagnostics are presented in Figs. 6, 7, 8, and 9.

Figure 6 shows the evolution over time of the α parameter of the Gamma-Gompertz model (see Eq. 1). This parameter is supposed to gauge the log-mortality at the beginning of the cohort’s mortality trajectory, which means, in our case, at age 80. The Sardinian and the Italian populations are characterized by a quite similar evolution in this parameter. Initial mortality was high at the beginning of the period under analysis, then it declines until about 1880, when mortality goes through a phase of stagnation which lasts for 15 to 20 years. In the last part of our series, the initial mortality parameter shows a new and more accentuated phase of decline. This second phase of mortality reduction seems to start earlier in Italy—with the cohort born between 1890 and 1895—and then in Sardinia, around 1900. Both these timings seem to be in line with the introduction of major legislative innovations: Crispi’s sanitary reform of 1888 and the law on the “Chinino di Stato” of 1900. According to our analysis, the Sardinian cohorts experienced, at the beginning of the period under observation, a higher mortality than the Italian cohorts. This is especially true for the Sardinian male cohorts. However, at the end of the period, the situation apparently reverted, with the Sardinian cohorts presenting lower initial mortality than their Italian equivalents. Caution is, though, needed in interpreting these results, because, the uncertainty (standard errors) connected to the estimates of the Sardinian cohort’s initial mortality is much higher than for Italy as a whole, as can be inferred by the larger confidence bands associated with the Sardinian estimates (see Fig. 6). This likely depends on the population of Sardinia being much smaller than the total Italian population, thus, entailing larger sample variance.

Figure 7 presents the results for the evolution of the β parameter of the Gamma-Gompertz model, the so-called rate of aging. Until the end of the 19th century, the rate of aging in Sardinia shows a stationary trend around very high values. Among females, the mean value of the rate of aging before the onset of the epidemiological transition was about 0.2. When males are considered, the mean value of the rate of aging is even higher, about 0.25. These estimates for the rate of aging appear to significantly diverge from the values of 0.1 (Baudisch and Vaupel 2010) or 0.14 (Rau cited in Missov 2010) proposed within the constant senescence hypothesis. Actually, to the best of our knowledge, the estimates of the rate of aging for the pre-transitional period in Sardinia are the highest yet recorded. In almost perfect coincidence with the large-scale distribution of quinine in Sardinia, the evolution of the rate of aging experienced a dramatic fall. At the end of the period of analysis (1908), the rate of aging drops to 0.07 among females and to the extremely low value of 0.02 among males. For males, the value attained by the rate of aging in 1908 was so low that it cannot even be considered as significantly different from zero.

From a theoretical point of view, the dramatic reduction in the rate of aging observed in those Sardinian cohorts born after 1899 could be due to period effects. In fact, the cohorts born after 1899 enter observation 80 years later, after several important medical innovations. At least theoretically, this process may have entailed a systematic underestimation of the rate of aging, by downward shifting of the terminal part of the cohort hazard function (see on that Salinari and De Santis 2014).

Even if it is not possible to completely exclude the existence of period effects at this stage of the analysis, it seems, nonetheless, implausible that such effects have played a decisive role in the decline of the Sardinian rate of aging, as observed for the beginning of the 20th century. The evolution of the rate of aging for the cohorts born after 1899 in Italy (Fig. 7) shows only a minor decline in the rate of aging starting from the 1890s. Hence, in order to explain the observed reduction in the Sardinian rate of aging by period effects, one has to assume that a particularly effective medical innovation was introduced into Sardinia, but not into the rest of Italy. However, this seems highly unlikely from a historical point of view.

It also seems wise to exclude the possibility that the deceleration of the speed of aging experienced by the cohorts that had large-scale access to quinine is a statistical artifact generated by the (spurious) correlation existing between the *α* and *β* parameters of the model. Actually, it is known that the *α* and *β* parameters of the Gompertz model are negatively correlated (Strehler and Mildvan 1960). This means that in those countries and epochs which present lower initial log-mortality (*α*) the rate of aging tends to be higher, and vice versa. The reason behind this phenomenon is still unclear, but some recent advances in this domain suggest that it may result from the “inherent statistical properties of the methods used to estimate the actuarial parameters [of the model]” (Burger and Missov 2016:36). Independently of the reasons for this result, it is important to stress that the correlation we actually observe between the α and β parameters is positive. In other words, the rate of aging of the Sardinian cohorts, and to a lesser extent, of the Italian cohorts, declines when the initial mortality of the cohorts is also declining. According to the Sthreler-Mildvan’s findings, we would have expected the opposite. We may therefore conclude that the correlation between the Gompertz parameters cannot explain the reduction in the rate of aging observed in Sardinia after 1900.

Thus, if period effects and the spurious parameters correlation can be reasonably ruled out, then the explanation of the decline in the rate of aging for the cohorts born after 1895, should be traced back to changes experienced by these cohorts in early life. The reduction in the rate of aging, in fact, occurs in those cohorts that had easier access to quinine, due to the 1900 law.

There are some clues as to the nature of the phenomenon responsible for the fall in the rate of aging in the cohort born at the beginning of the 20th century. These come from the observation of the temporal evolution of the third parameter of the Gamma-Gompertz model, the initial variance of frailty. This parameter measures how heterogeneous cohorts are at 80 years. Prior to the Sardinian epidemiological transition and the introduction of quinine, the initial variability of frailty was much higher in Sardinia than in Italy (six or seven times higher). According to this result the Sardinia cohort were, thus, much more heterogeneous than Italy as a whole. This may be consistent with a situation where most of the population suffered from malaria. What is particularly interesting, however, is that the temporal evolution of the heterogeneity of the Sardinian cohorts follows exactly the same pattern as the rate of aging. At the very end of the 19th century, the value of this parameter suddenly drops to very low levels.

In the last 2–3 years of our observation window, among males, where the drop was more dramatic, the initial variance of frailty becomes negative. The same phenomenon emerges in the Italian male cohorts during the 1890s. This represents a violation of one of the central assumptions of the Gamma-Gompertz model, because, by construction, the initial variability of frailty cannot be less than zero. This result suggests that the Gamma-Gompertz model is not able to consistently capture the evolution of mortality with age during a period characterized by a dramatic reduction in the disease-load suffered in their early stages of life by the members of cohorts. In the last section of this paper, we will try to convince the reader that this failure of the Gamma-Gompertz is consistent with the inflammation theory.

Another clue to the problem inherent in the Gamma-Gompertz framework, comes from diagnostic tests. In particular, Fig. 9 shows the *p* values associated with the Shapiro-Wilk normality test applied to the residuals of our Poisson regressions. In most cases, the Shapiro-Wilk test does not reject the null hypothesis of normality (*p* value > 0.05). However, for the cohorts born in the early years of the 20th century in Sardinia, the first that were exposed to quinine, the test frequently rejects the hypothesis of normality. This constitutes a second indication that the Gamma-Gompertz model may not be able to fully capture the mortality trajectory followed by the Sardinian cohorts born at the turn of the 20th century.

The analysis performed seems to exclude the possibility that these incoherencies stem from period effects or parameters correlation. As anticipated in the methodology section, to exclude this possibility in a more formal way, we have first differentiated, with respect to age, the log mortality hazard functions of the cohorts that form our dataset. To test if the Sardinian cohorts went through a significant change in their rate of aging after the onset of the epidemiological transition, two models have been run on the differentiated hazards. In the first one, the mean rate of aging for all cohorts under observation has been estimated trhough Eq. (5). In the second, five different rates of aging have been estimated according to the different decades of birth of the cohorts (Eq. 6). The same analysis has been repeated for Italy and Sardinia, separately for males and females. The results of this analysis are shown in Table 2. Table 2 confirms that the decline in the rate of aging that we observed among the Sardinian cohorts is statistically significant, despite the noise which inevitably affects the estimates of Gamma-Gompertz parameters in such a small population. Both male and female cohorts born in the first decade of the 20th century thus show significantly lower rates of aging with respect to older cohorts. To test the robustness of our results we repeated this analysis using a Poisson regression and the introduction of time fixed effects in the model (results not shown). This second analysis confirmed that the decline in the rate of aging persists after controlling for period effects.

**Table 2:** Testing the constant senescence hypothesis

	Sardinia - males		Sardinia - females		Italy - males		Italy - females	
	Estimate	SE	Estimate	SE	Estimate	SE	Estimate	SE
Equation 5								
Rate of aging								
All cohorts	0.19	0.016	0.18	0.013	0.097	0.006	0.11	0.081
σ^2^ (mean value)	[0.98]	–	[0.81]	–	[0.10]	–	[0.14]	–
*R*^2^	0.11	–	0.12	–	0.02	–	0.05	–
Equation 6								
Rate of aging								
Cohorts 1860–1869	0.35	0.107	0.25	0.072	–	–	–	–
Cohorts 1870–1879	0.22	0.037	0.20	0.035	0.13	0.014	0.12	0.014
Cohorts 1880–1889	0.21	0.034	0.20	0.028	0.11	0.011	0.12	0.010
Cohorts 1890–1899	0.22	0.029	0.23	0.023	0.10	0.012	0.11	0.009
Cohorts 1900–1908	0.10	0.031	0.09	0.023	0.08	0.01	0.10	0.007
σ^2^ (mean value)	[1.07]	–	[0.91]	–	[0.12]	–	[0.15]	–
*R*^2^	0.12	–	0.15	–	0.04	–	0.06	–
ANOVA	*F* stat.	Pr(> *F*)	*F* stat.	Pr(> *F*)	F stat.	Pr(> *F*)	F stat.	Pr(> *F*)
	2.92	0.02	5.82	< 0.001	3.53	0.01	0.71	0.55

*Source*: Authors’ calculations on HMD data

Table 2 also confirms that the evolution of the rate of aging in Italy shows some relevant differences with respect to Sardinia. The decline in the rate of aging seems to start earlier in Italy—in the 1890s—and the magnitude of the decline is much smaller than in Sardinia. This decline appears to be statistically significant for the male, but not for the female cohorts. The latter result may be a consequence of the statistical techniques used to perform the test. As we have seen, differentiation entails several advantages, but it does not come without costs: it doubles the random noise in our data, as is confirmed by the low values of *R*^2^. The low level of this coefficient does not indicate, in the present context, poor performance in the model. Rather, it indicates that the random noise affecting the data is high because of differentiation. This noisiness in our data probably ends up reducing the power of the test on the temporal change of the rate of aging. Likely for this reason, the small reduction in the rate of aging experienced by the female Italian cohorts from the 1890 to 1908 is not statistically significant.

We may now wonder whether, before the onset of the epidemiological transition, Italy and Sardinia presented significantly different rates of aging as Fig. 7 seems to suggest. To test this hypothesis, we pooled together the Italian and the Sardinian differentiated log-hazard functions for the cohorts born before the onset of the epidemiological transition. For Italy, we thus considered the cohorts born between 1872 and 1888. For Sardinia instead, we selected the cohorts born between 1866 and 1899. We first estimated a model with a single rate of aging for the Italian and the Sardinian population, and then we compared this model via an F-test on a second model, where the Italian and the Sardinian cohorts can have different rates of aging (see Table 3). The test confirms that before the onset of the epidemiological transition and the introduction of quinine, the rate of aging among the Sardinian cohorts was significantly higher than that of the Italian cohorts.

**Table 3:** Comparison between the Italian and the Sardinian rate of aging before the onset of the epidemiological transition

	Males		Females	
	Estimate	SE	Estimate	SE
Equation 5				
Rate of aging				
All cohorts	0.12	0.007	0.12	0.006
σ^2^ (mean value)	[0.42]	–	[0.38]	–
*R*^2^	0.057	–	0.057	–
Equation 7				
Rate of aging				
Italy	0.12	0.070	0.12	0.006
Sardinia	0.21	0.041	0.22	0.035
σ^2^ (mean value)	[0.83]	–	[0.76]	–
*R*^2^	0.063	–	0.065	–
ANOVA	*F* stat.	Pr(> *F*)	F stat.	Pr(> *F*)
	6.12	0.010	7.91	0.005

*Source*: Authors’ calculations on HMD data

## Discussion

The application of the Gamma-Gompertz model to the analysis of the Italian and Sardinian lifetables before and during the epidemiological transition has allowed us to present three main results. First, we found that before the epidemiological transition, Sardinia had a much higher rate of aging than Italy. Second, we found that, in almost perfect coincidence with the onset of the epidemiological transition, a dramatic fall in the rate of aging occurred. Third, we noticed, both for the Italian and the Sardinian male cohorts, that as the epidemiological transition took place, the estimates of the initial variance of frailty produced by the Gamma-Gompertz model occasionally became negative. The last result represents a clear violation of the general theoretical framework from which the Gamma-Gompertz model itself was derived. How should we interpret these results in the light of the three theories of aging outlined at the beginning of this paper?

First, it must be noted that the results presented seem to suggest that at least one of the main postulates underpinning the Gamma-Gompertz model—the constant senescence hypothesis—is problematic. The Gamma-Gompertz model belongs, indeed, to the wider class of the proportional hazard models, where covariates are supposed to affect the hazard function in a multiplicative way. In such a framework the individual log hazard functions can, thus, be shifted upward or downward by the effect of a certain covariate, but they cannot change their slope (i.e., the rate of aging).

Nevertheless, we found evidence that different populations do present hazard functions with significantly different slopes. This was apparent both from a geographic comparison—the rate of aging was much higher in Sardinia than in Italy before the onset of the epidemiological transition—and from a temporal comparison—the rate of aging falls dramatically in the last cohorts of our series. These results highlight a potential contradiction between one of the constitutive hypotheses of the Gamma-Gompertz model—the constant senescence hypothesis—and the evolution of mortality in Sardinia and Italy.

With regards to Sardinia, the reduction in the rate of aging in an epoch characterized by a rapid reduction in the infective load (probably due to quinine) appears to be consistent, at least at first sight, with the inflammation theory. The inflammation theory seems also to be supported by several recent analyses conducted on the present-day Sardinian population, which have revealed a high prevalence of male centenarians, especially in the central part of the island (the so-called blue zone; Poulain et al. 2013). At the 2001 Census there were in Sardinia 1.2 centenarians for every 10,000 inhabitants with a female-to-male ratio of 2.5/1, among the lowest ever recorded (Caselli et al. 2006).

Most of these centenarians were born in the last cohorts analyzed in this paper. The same cohorts that present a very low rate of aging. However, caution is needed on this point. In particular, one may argue that if the Gamma-Gompertz model is not the most appropriate for describing the evolution of mortality during the epidemiologic transition, then the estimates of the rate of aging produced with this model may be biased.

**Fig. 10:**
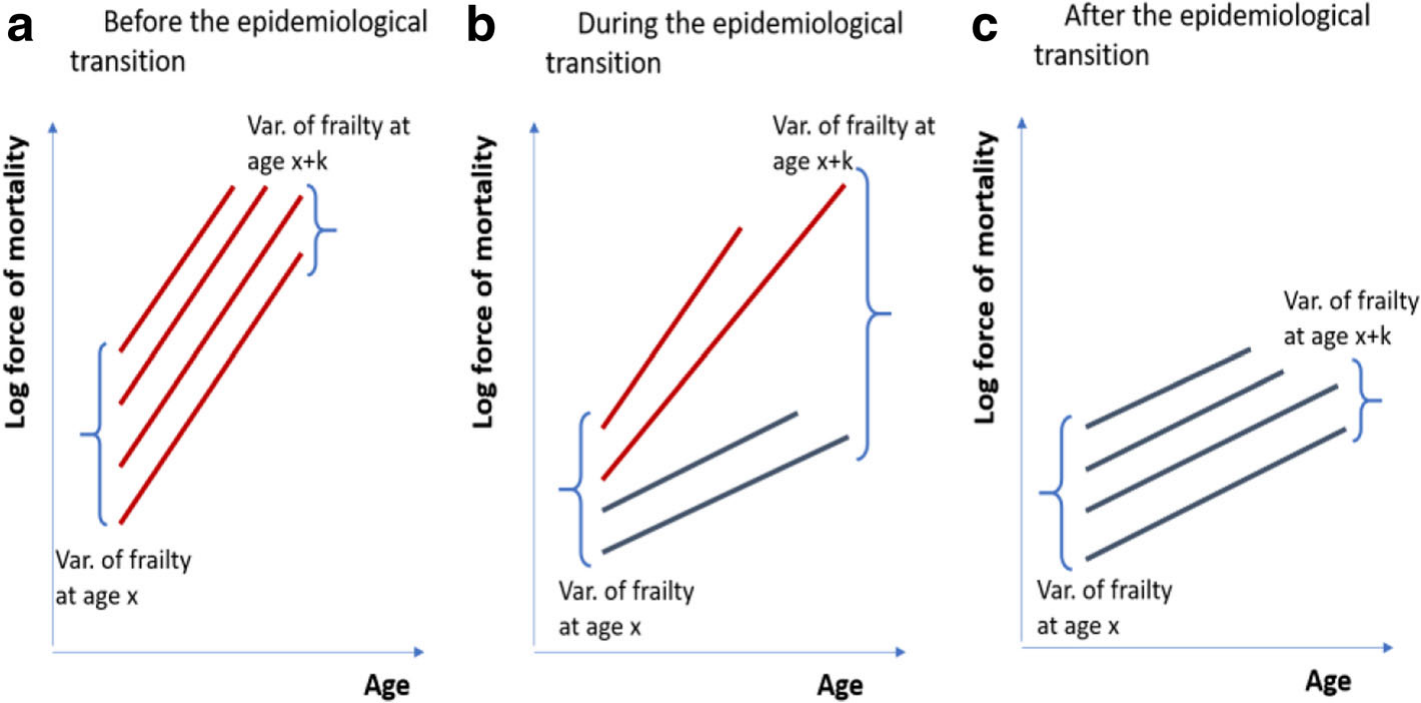
Evolution of the rate of aging according to the inflammaging theory before (Panel **a**), dunring (Panel **b**) and after (Panel **c**) the epidemiological transition. *Note*: the segment in the three panels represent individual log hazard functions

Some evidence for the inflammation theory comes, however, from the failure of the Gamma-Gompertz model in male cohorts during the onset of the epidemiological transition. After all, this is what one would expect if the inflammation theory were correct. Let us assume, indeed, that the members of a cohort, as claimed by the inflammation theory, can be characterized by different rates of aging according to the disease load suffered during their early years. Before the onset of the epidemiological transition, we would thus expect, as shown in Fig. 10a, that most individuals in Sardinia would have faster aging, because of the heavy exposure to diseases during their infancy. Similarly, at the end of the epidemiological transition, as shown in Fig. 10c, we would expect that most of individuals would be characterized by slower aging, given that the introduction of vaccines, antibiotics, and quinine drastically reduced the disease load experienced during childhood. In between these two phases, however, we might imagine the existence of an intermediate phase (Fig. 10b) during which the cohorts have a mixture of individuals. Some of them have not yet benefitted from medical innovations, and are, thus, exposed to a heavier disease load during their infancy and, because of that, they present higher rate of aging. Others have gained access to medical innovations earlier and, therefore, suffer a lighter disease load, which implies a slower rate of aging. In this intermediate situation, the hazard rates of the individuals diverge producing a progressive rise in the variance of frailty. This rise is not compatible with the Gamma-Gompertz model, which is built on the idea that the variance of frailty decreases with age because of selection. The inflammation theory seems, in a way that is consistent with our results, able to predict the failure of the Gamma-Gompertz model during the more intense phase of epidemiological transition.

Furthermore, the very low levels of nutrition observed in Sardinia, coupled with the dramatic fall off in the disease burden in the last years of the 19th century, might help to explain why the decline in the Sardinian rate of aging has been so dramatic compared with other European regions. However, the inconsistencies that come out in the application of the Gamma-Gompertz model to the Sardinian data, counsel caution on this point.

The explanations advanced in the literature to justify the high prevalence of male centenarians in Sardinia have emphasized the role played by genetic factors. The particular historical evolution followed by the Sardinian population seems to spontaneously suggest this direction of research. In fact, important parts of the island remained relatively isolated, at least until the Unification of Italy (1861): Gatti and Puggioni (1988) believe, meanwhile, that the breakdown of the “genetic isolates” took place even later, in the 1950s. The possibility that genetic factors played a role in the evolution of the rate of aging in Sardinia cannot be entirely ruled out then. However, the analysis presented in this paper suggests that the very low Sardinian rate of aging at the beginning of the 20th century may depend on other factors like nutrition and disease load.
